# The Mediating Role of Work Engagement in the Effect of Workplace Incivility on Quiet Quitting: A Cross‐Sectional Study in Healthcare Workers

**DOI:** 10.1155/jonm/5144085

**Published:** 2026-10-23

**Authors:** Hafize Boyacı, Emin Aksan, Selma Söyük

**Affiliations:** ^1^ Cerrahpasa Medical Faculty Fikret Biyal Central Laboratory Istanbul University-Cerrahpasa Istanbul Türkiye istanbul.edu.tr; ^2^ Department of Medical Services and Techniques Medical Documentation and Secretarial Program Istanbul Kultur University Istanbul Türkiye iku.edu.tr; ^3^ Department of Health Management Faculty of Health Sciences Istanbul University-Cerrahpasa Istanbul Türkiye istanbul.edu.tr

**Keywords:** healthcare workers, mediation analysis, nursing management, quiet quitting, work engagement, workplace incivility

## Abstract

**Background:**

Workplace incivility is common in healthcare settings and negatively affects employee attitudes and workforce stability, yet its impact on work engagement and quiet quitting remains underexplored.

**Aim:**

To investigate the mediating role of work engagement in the effect of workplace incivility on quiet quitting intention among healthcare workers.

**Methods:**

Cross‐sectional descriptive study. Data were collected between December 2025 and January 2026 from 278 healthcare professionals (managers, physicians, nurses, technicians and administrative staff) at a private hospital in Istanbul, using convenience sampling.

**Results:**

Workplace incivility correlated positively with quiet quitting (*r* = 0.388, *p* < 0.001) and negatively with work engagement (*r* = −0.274, *p* < 0.001); work engagement correlated negatively with quiet quitting (*r* = −0.518, *p* < 0.001). Work engagement partially mediated the association between incivility and quiet quitting (unstandardised indirect effect *B* = 0.123, 95% CI [0.068, 0.188]; completely standardised indirect effect = 0.122, 95% CI [0.067, 0.186]); the direct effect remained significant (*β* = 0.266, *p* < 0.001).

**Conclusions:**

Workplace incivility was associated with higher quiet quitting intention, both directly and indirectly through lower work engagement. Because the design was cross‐sectional, these findings describe associations rather than causal effects.

**Implications for Nursing Management:**

Nurse managers should treat workplace incivility as a modifiable organisational risk factor for quiet quitting rather than an interpersonal nuisance. Zero‐tolerance policies, accessible reporting mechanisms and civility training can reduce exposure to disrespectful behaviour. Because work engagement partially buffers this relationship, managers should also invest in engagement‐building strategies such as recognition programmes, participatory decision‐making, supportive supervision and adequate staffing. Routine monitoring through staff surveys can identify early warning signs before disengagement affects patient care and retention.

**Practitioner Points**
• Healthcare workers who experienced workplace incivility reported lower work engagement and higher quiet quitting intention.
• Work engagement partially mediated the relationship between workplace incivility and quiet quitting intention.
• Routine engagement monitoring and early screening for quiet quitting signals can guide timely intervention, protecting both staff wellbeing and patient care quality.

## 1. Introduction

Workplace incivility (WI), although described as low intensity in the literature, is considered one of the negative behaviours with significant and far‐reaching consequences. The concept, first developed by Andersson and Pearson [1], was defined as behaviour characterised by the violation of workplace norms of mutual respect, usually carried out with ambiguous intent. In this respect, WI is considered distinct from direct forms of aggression, yet as a condition that prepares the ground for such behaviour over time. Cortina et al. [2] positioned WI as a subdimension of behaviours such as aggression, bullying, petty tyranny and workplace violence. Encompassing rudeness, humiliation, belittlement and disrespect, WI is costly for both individuals and organisations. The theoretical basis of this study is conservation of resources (COR) theory. Developed by Hobfoll [3], COR theory proposes that individuals seek to acquire, protect and develop the resources they possess. Resources such as psychological safety, being treated with respect, emotional energy, motivation and social support are among the most important personal resources for employees at work [3, 4]. According to COR theory, negative experiences in the work environment cause employees to lose resources, which in turn reduces their energy and their engagement with work. COR theory therefore provides an appropriate theoretical framework for explaining the relationships among WI, work engagement (WE) and quiet quitting (QQ).

WE, regarded as one of the positive organisational behaviours underpinning organisational efficiency and effectiveness, is the state most directly affected by this process. Kahn [5] defined engagement as an individual’s physical, cognitive and emotional attachment to their work, while Schaufeli et al. [6] characterised it through vigour, dedication and absorption, emphasising a positive and sustainable attachment to work. Engagement is particularly consequential for healthcare workers in coping with demanding tasks, and organisational factors such as a supportive workplace climate play an important role in sustaining it in healthcare settings [7].

Another behavioural pattern that emerges in this context is QQ, characterised by employees maintaining their attachment to their work at a limited level. This concept refers to employees limiting their efforts to the minimum by avoiding responsibilities beyond their job descriptions [8].

It describes behaviour in which individuals avoid additional responsibilities beyond their job description, minimise their effort at work and refrain from tasks they are not required to perform. While this attitude may be perceived at the individual level as a strategy for establishing work–life balance, from an organisational perspective, it may be interpreted as resistance to work [9].

The transformations brought about by the pandemic have altered employees’ perspectives on their work, triggering the development of different ways of thinking and the concept of QQ. Factors such as high stress, increased workload and burnout, particularly in healthcare institutions, are reported to have prepared the ground for the spread of QQ [10]. Riehle et al. [11] addressed QQ as a factor weakening WE among service‐sector employees. Unlike conventional resignation, QQ refers not to physical departure from the organisation but to a decline in performance and productivity [12]. Gün [13] reported that the main criterion most strongly influencing QQ behaviour was economic factors.

Understanding the relationship among WI, WE and QQ will be beneficial for both healthcare workers and healthcare managers. Although recent studies [10, 14, 15], including Stankovic and Slavkovic [15], have begun to identify the individual and work‐related antecedents of QQ among healthcare workers, the role of WI as a predictor of QQ and the mediating role of WE in this relationship remain insufficiently examined. The present study therefore investigates the potential mediating role of WE in the effect of WI on QQ among healthcare workers. As a persistent stressor, WI depletes employees’ psychological resources; the resulting decline in WE leads employees to withdraw from organisational role behaviours and to develop a tendency towards QQ, which is a resource conservation strategy consistent with the predictions of COR theory [3, 4].

Because COR theory defines WE as the resource‐based psychological state most directly depleted by stressors such as incivility, examining its mediating role provides a theoretically grounded explanation of how and why WI translates into QQ among healthcare workers. This explanation differs from previous studies that focused on burnout, quality of working life or moral resistance.

Accordingly, the following hypotheses were tested:

 H1. WI is positively associated with QQ intention among healthcare workers.
 H2. WE is negatively associated with QQ intention among healthcare workers.
 H3. WE mediates the association between WI and QQ intention.

## 2. Materials and Methods

This study was designed as a descriptive, cross‐sectional study. The data were collected between December 2025 and January 2026.

### 2.1. Research Objective and Significance

The aim of this study is to investigate the mediating role of WE in the effect of WI on the intention to resign silently among healthcare workers, and to shed light on the relationships between WI, the intention to resign silently and WE. The role of the WE variable in the relationship between WI and QQ intention will be examined. By treating the WI experienced by healthcare workers as a social phenomenon, the study will examine the relationship between this behaviour, QQ intentions and WE, thereby seeking to fill a gap in the literature on this subject. A quantitative research method was used in the study. The study is significant in terms of raising awareness among relevant staff and managers and contributing to the literature.

### 2.2. Measures

The data were collected using a questionnaire. The questionnaire consisted of a total of 21 items covering WI (7), WE (3) and intention to resign silently (7), as well as demographic and descriptive information (4). The questionnaire was administered using the primary data collection methods of online and face‐to‐face surveys. The study was designed using scales that had previously been employed in the literature and were considered to have high reliability. The characteristics of the measurement tools used in the study are as follows:

WI Scale: The seven‐item WIS developed by Cortina et al. [2] was used to measure perceptions of WI. The scale was adapted into Turkish by Gök [16]. The items on the scale were designed according to a 5‐point Likert scale (*Never*–*Always*) [16].

QQ Scale: QQ is a passive form of resistance where an employee exerts only enough effort to fulfil the duties specified in their job description and displays reluctance towards their tasks. The QQ Intention Scale was developed by Anand et al. [17] and adapted into Turkish by Yıldız et al. [18], who reported a unidimensional structure and a Cronbach’s alpha of 0.82. The scale consists of seven items rated on a five‐point Likert scale ranging from ‘*strongly disagree*’ to ‘*strongly agree*’. All seven items were administered in the present study, but two items were removed following factor analysis. The analysis was conducted with the remaining five items.

WE Scale: The Utrecht Work Engagement Scale (UWES) was originally developed by Schaufeli et al. [6] as a 17‐item instrument and subsequently shortened to nine items [19]. The Turkish adaptation of the UWES‐9 was carried out by Eryılmaz and Doğan [20] and by Özkalp and Meydan [21]. The scale comprises three subdimensions of three items each: vigour, dedication and absorption. The present study used the ultra‐short three‐item version (UWES‐3), which contains one indicator of each subdimension.

The Turkish form of the UWES‐3 was obtained from the researchers who conducted the Turkish adaptation. Its psychometric properties in Turkish were established by Güler et al. [22], who validated the three‐item version in a Turkish sample and reported acceptable confirmatory fit for the single‐factor model. The response format is the six‐point frequency scale of that validated Turkish version, ranging from 1 (‘*Almost never*/*A few times a year or less*’) to 6 (‘*Always*/*Every day*’). No modification or rescaling of the item wording, response format or scoring procedure was carried out by the present authors. Cronbach’s alpha was 0.85.

### 2.3. Study Population and Sample

The study population comprised 620 healthcare workers employed at a private hospital operating in Istanbul. Data were collected between December 2025 and January 2026 using convenience sampling; this method was preferred because of the demanding work schedules of healthcare workers and the limited time available during the data collection process. Inclusion criteria were (i) being actively employed at the hospital during the data collection period, (ii) participating in the study voluntarily and (iii) being a manager, physician, nurse, healthcare technician/technologist or administrative staff member. Exclusion criteria were (i) questionnaires that did not contain usable responses and (ii) questionnaires in which the participant’s reported occupation could not be assigned to one of the five predefined occupational groups (manager, physician, nurse, health technician/technologist, administrative staff) used in the analyses. The data collection process was conducted in accordance with the principles of the Declaration of Helsinki.

From a target population of 620 employees, 405 questionnaires were returned, resulting in a response rate of 65.3%. Five questionnaires lacking usable responses were excluded, leaving 400 records for further analysis. 122 participants who selected ‘other’ for the occupation question and could not be reliably assigned to one of five predefined occupational groups were excluded from the analysis. Thus, the final analytical sample consisted of 278 participants who were managers, physicians, nurses, health technicians/technologists or administrative staff; this number corresponds to 68.6% of the returned questionnaires and 44.8% of the 620 eligible employees. Data were collected using the same questionnaire both online (Google Forms) and face‐to‐face. The online questionnaire link was sent to participants through electronic communication channels, and printed questionnaires were administered face‐to‐face by the researchers. Responses obtained from the printed questionnaires were transferred to electronic form using the same variable coding as Google Forms, and all data were consolidated into a single dataset. Before data cleaning, possible duplicate participation was examined by comparing records and demographic information, and no duplicate entries were found.

No power analysis was conducted before data collection to assess the statistical adequacy of the sample size. However, a sensitivity (post hoc) power analysis was carried out after data collection using G∗Power 3.1. For two‐tailed Pearson correlations (*α* = 0.05), a sample of 278 was found to detect a correlation of *r* = 0.20 with 92% power and a correlation of *r* = 0.15 with 71% power. In addition, 258 participants were calculated to be sufficient to detect an effect of *r* = 0.20 with 90% power.

### 2.4. Ethics Statement

Ethics committee approval for the study was obtained from the Ethics Committee for Social and Human Sciences Research of Istanbul University‐Cerrahpaşa (04 November 2025, approval number 2025/608). Informed consent was obtained from the healthcare workers who agreed to participate in the study, and the confidentiality and anonymity of their responses were guaranteed. Data were collected in accordance with the Declaration of Helsinki.

### 2.5. Data Analysis

The statistical analyses of the study were performed using IBM SPSS Statistics 31.0 (IBM Corp., Armonk, NY, USA), Lisrel 8.8. and Hayes PROCESS macro v4.2 (Model 4). The assumption of normal distribution was assessed by examining skewness and kurtosis values [23]. Missing data were examined in detail before the main analyses. Of the 278 participants, 262 (94.2%) answered all items completely. Item‐level missingness ranged from 0.00% to 2.52% and did not exceed 5% for any item (WI 0.57%; WE 0.24%; QQ intention 1.54%). Little’s missing completely at random (MCAR) test was applied to evaluate the missing‐data mechanism, and the results indicated that the values were MCAR, *χ* ^2^ (161) = 150.06 and *p* = 0.721. Scale‐level MCAR tests were likewise nonsignificant (WI *χ* ^2^ (39) = 18.33, *p* = 0.998; QQ intention *χ* ^2^ (14) = 9.69, *p* = 0.784; WE *χ* ^2^ (2) = 2.60, *p* = 0.272). Because the rate of missing data was low and the MCAR assumption was met, missing observations were completed using mean substitution and all analyses were conducted on the completed dataset.

Because all variables were collected from the same participants at a single time point using self‐report methods, common method bias was assessed. Procedurally, participant anonymity and confidentiality were ensured, the scales were presented under separate headings, and different response formats were used. Statistically, Harman’s single‐factor test yielded four factors with eigenvalues greater than 1, and the first factor accounted for 34.62% of the total variance. Furthermore, the single‐factor confirmatory model showed poor fit (CFI = 0.643; RMSEA = 0.148), whereas the hypothesised three‐factor model provided acceptable fit (CFI = 0.938; RMSEA = 0.063). Considered together with the moderate correlations among the constructs (|*r*| = 0.274–0.505), these results indicate that common method bias did not substantially affect the findings.

Exploratory factor analyses were conducted to assess the construct validity of the scales, and Cronbach’s alpha coefficients were computed to assess their internal consistency. Participants’ demographic characteristics and the study variables were summarised using means, standard deviations, frequencies and percentages. Relationships among the variables were examined using Pearson correlation analysis. The mediating role of WE in the relationship between WI and QQ intention was tested using the Hayes PROCESS macro v4.2 (Model 4). The significance of indirect effects was evaluated with 95% confidence intervals based on 5000 bootstrap resamples [24], and the level of statistical significance was set at *p* < 0.05. Items were retained on the basis of criteria specified in advance (factor loading ≥ 0.40; corrected item‐total correlation ≥ 0.30).

## 3. Results

Participants’ demographic characteristics are presented in Table 1. The majority of the sample were women (68.0%), aged 18–34 years (61.2%) and educated to bachelor’s degree level (62.6%). Detailed demographic distributions are given in Table 1.

**TABLE 1:** Participant characteristics (*N* = 278).

Variable		Frequency (*n*)	Per cent (%)
Gender	Female	189	68.0
	Male	89	32.0

Age	18–34 years	170	61.2
	35–44 years	65	23.4
	45–54 years	34	12.2
	55 years and over	9	3.2

Education	Secondary school or equivalent	56	20.1
	Bachelor’s degree	174	62.6
	Master degree	34	12.2
	Medical Speciality Certification	14	5.0

Occupation	Managers	35	12.6
	Doctor	18	6.5
	Nurse	72	25.9
	Healthcare professional/technician	82	29.5
	Administrative staff	71	25.5

Total		278	100.0

### 3.1. Preliminary Findings

Prior to evaluating the model, preliminary analyses were conducted, including descriptive statistics, reliability measures, an examination of the normality assumption and the calculation of correlation coefficients. It was determined that the data were normally distributed ± 1.5 [23]. The factor structure of the scales was examined.

WI: KMO 0.906; Bartlett’s sphericity test: *χ* ^2^ (21) = 1155.66; Sig. = 0.001 < 0.05. It was found to have a single‐factor structure and to explain 64.73% of the total variance. Factor loadings range from 0.721 to 0.867. Cronbach’s alpha coefficient was 0.908.

QQ: The psychometric properties of the scale were re‐examined in the current sample because the Turkish adaptation is new. As a single component was expected, extraction was performed by principal component analysis without rotation. Item retention was evaluated against criteria specified in advance: a factor loading of at least 0.40 and a corrected item‐total correlation of at least 0.30; communalities were examined as a supporting indicator.

Sampling adequacy was found to be satisfactory (KMO = 0.776), and the correlation matrix was suitable for factor analysis (Bartlett’s test of sphericity *χ* ^2^ (21) = 364.55, *p* < 0.001). A single component accounted for 38.89% of the total variance; Cronbach’s alpha was 0.720, and McDonald’s omega was 0.731. Two items failed the prespecified criteria. QQ2 (‘I do the minimum required to keep my position’) showed a factor loading of 0.326, a communality of 0.056 and a corrected item‐total correlation of 0.223, all below the respective thresholds, and its deletion increased alpha from 0.720 to 0.736. The exclusion of QQ2 has a conceptual implication. QQ2 reflects limiting effort to the minimum required by the job, a commonly recognised behavioural feature of QQ. Its poor performance may indicate that behavioural disengagement is not adequately captured in healthcare settings, where formal role requirements are clearly defined. The retained five items primarily reflect the attitudinal dimension of QQ. Thus, the construct measured here is better understood as attitudinal disengagement rather than the full behavioural expression of QQ, which is acknowledged as a limitation. QQ7 (‘I feel my supervisor does not value me’) also fell below the criteria, with a communality of 0.116 and a corrected item‐total correlation of 0.285.

In line with these results, QQ2 and QQ7 were excluded and all subsequent analyses were conducted with the remaining five items. In the five‐item solution, the structure was unidimensional (a single eigenvalue greater than 1), sampling adequacy remained satisfactory (KMO = 0.785; Bartlett’s test of sphericity *χ* ^2^ (10) = 298.26, *p* < 0.001), and the single component accounted for 50.33% of the total variance. Factor loadings ranged from 0.541 to 0.791 and corrected item‐total correlations from 0.362 to 0.602, with all items meeting the prespecified criteria. Internal consistency improved (Cronbach’s alpha = 0.746; McDonald’s omega = 0.754), and the mean inter‐item correlation was 0.373. Item‐level statistics for all seven items, together with the retention decision for each, are presented in Table 2.

**TABLE 2:** Factor loadings, communalities and item statistics for the quiet quitting intention scale (*N* = 278).

Item	Content	Loading	*h* ^2^	M (SD)	Corrected item‐total r	*α* if item deleted	Decision
QQ1	I avoid extra work when there is no additional pay	0.567	0.203	2.75 (1.31)	0.424	0.689	Retained
*QQ2*	*I do the minimum required to keep my position*	*0.326*	*0.056*	*2.62 (1.24)*	*0.223*	*0.736*	*Excluded*
QQ3	I feel a lack of learning and development opportunities	0.699	0.384	3.06 (1.27)	0.495	0.672	Retained
QQ4	I feel a lack of meaningfulness in my work	0.704	0.386	2.71 (1.26)	0.509	0.668	Retained
QQ5	I am unwilling to attend meetings at my institution	0.755	0.501	2.73 (1.20)	0.548	0.658	Retained
QQ6	I lack passion and enthusiasm to work beyond requirements	0.739	0.455	2.87 (1.21)	0.554	0.657	Retained
*QQ7*	*I feel my supervisor does not value me*	*0.441*	*0.116*	*2.84 (1.34)*	*0.285*	*0.722*	*Excluded*

*Note:* Extraction: principal component analysis, unrotated single‐factor solution; 38.89% of variance explained; KMO = 0.785; Bartlett’s test of sphericity *χ* ^2^(10) = 298.26, *p* < 0.001.

Confirmatory factor analysis supported this decision. The single‐factor model fitted the five‐item version well, *χ* ^2^ (5) = 10.25, *χ* ^2^/df = 2.05, CFI = 0.982, TLI = 0.964, RMSEA = 0.062 (90% CI [0.000, 0.116]) and SRMR = 0.030, whereas the same model fitted the original seven‐item version only marginally, *χ* ^2^ (14) = 42.72, *χ* ^2^/df = 3.05, CFI = 0.917, TLI = 0.876, RMSEA = 0.086 (90% CI [0.057, 0.116]) and SRMR = 0.056. Composite reliability (CR) rose from 0.729 to 0.753, and average variance extracted (AVE) from 0.300 to 0.387. The factor loadings of the scale are presented in Table 2.

WE: The psychometric properties of the UWES‐3 were re‐examined in the present sample. Exploratory factor analysis indicated that the scale has a single‐factor structure accounting for 75.53% of the total variance (KMO = 0.678; Bartlett’s test of sphericity *χ* ^2^ (3) = 360.62, *p* < 0.001). Factor loadings ranged from 0.817 to 0.916, communalities from 0.446 to 0.896, and corrected item‐total correlations from 0.620 to 0.788. Cronbach’s alpha was 0.838, CR 0.845 and AVE 0.650. These values exceed accepted psychometric thresholds and are consistent with the Turkish validation findings reported by Güler et al. [22] and the international validity results reported by Schaufeli et al. [25], supporting the UWES‐3 as a reliable and unidimensional instrument for measuring WE in this sample. The Turkish version of the UWES‐3 validated by Güler et al. [22] was used, and no modification or rescaling was made to the item structure, response format or scoring procedure of the scale. In addition, the Cronbach’s alpha coefficients of the WI, WE and QQ intention scales were 0.908, 0.838 and 0.720, respectively. All preliminary findings are presented in Table 3.

**TABLE 3:** Mean, standard deviation, skewness, kurtosis, alpha and correlation values for the research variables.

	*N*	Possible range	M ± SD	Skewness (SE)	Kurtosis (SE)	*α*	WI	WE	QQ
WI	278	1–5	1.96 ± 0.85	0.74 (0.15)	−0.20 (0.29)	0.908	—	−0.274^∗∗^	0.388^∗∗^
WE	278	1–6	4.50 ± 1.21	−0.61 (0.15)	−0.51 (0.29)	0.838		—	−0.518^∗∗^
QQ	278	1–5	2.83 ± 0.85	0.07 (0.15)	−0.34 (0.29)	0.746			—

*Note: N* = 278. M, mean; *α*, Cronbach’s alpha coefficient.

Abbreviations: QQ, quiet quitting; SD, standard deviation; SE, standard error; WE, work engagement; WI, workplace incivility.

^∗∗^ *p* < 0.001.

There are significant relationships among the variables included in the study: WI, QQ and WE. Healthcare workers employed at the healthcare institution where the study was conducted were asked whether they had been exposed to rude behaviour exhibited by managers or colleagues in the past year. The overall mean score for WI was found to be 1.96 ± 0.85, indicating a below‐average level. The WE scale used in the study measures healthcare workers’ levels of energy, commitment and immersion in their work. As the scale’s average score increases, it indicates that participants are more engaged in their work and demonstrate physical, cognitive and emotional dedication and commitment to their jobs. In the study, the average WE score was 4.50 ± 1.212, indicating an upper‐middle level. The mean QQ score, based on the five‐item version of the scale, was 2.83 ± 0.85, indicating a middle level. Relationships between variables were examined using Pearson correlation analysis. For this purpose, the values *r* = 0.10, *r* = 0.30 and *r* = 0.50 were used to estimate the magnitude and strength of the correlation, as proposed by Cohen [26]. WI was positively and significantly related to QQ intention (*r* = 0.388, *p* < 0.001) and negatively related to WE (*r* = −0.274, *p* < 0.001); WE was negatively and significantly related to QQ intention (*r* = −0.518, *p* < 0.001). The results of the correlation analysis are presented in Table 3.

Difference tests (independent‐sample *t*‐test and one‐way ANOVA) revealed no statistically significant differences between the demographic variables of gender, age, education and occupational status and the study’s main variables (WI, WE and QQ intention).

### 3.2. Mediation Analyses

The mediation analysis was conducted using the HAYES PROCESS v4.2 macro (Model 4), with WI entered as the independent variable, QQ as the dependent variable and WE as the mediating variable. To distinguish full from partial mediation, the significant effect of WI on QQ was evaluated with WE in the model: had this effect become nonsignificant, WE would have acted as a full mediator; because the direct effect remained significant while the indirect effect through WE was also significant, WE acts as a partial mediator. Full standardised and unstandardised path coefficients, together with the direct, indirect and total effects and their bootstrap confidence intervals, are reported in Tables 4 and 5 and are not restated here (Figure 1).

**TABLE 4:** Unstandardised and standardised coefficients for the mediation model.

Path	*β*	*B*	SE	*t*	*p*	95% CI for *B*	Model summary
WI ⟶ WE	−0.274	−0.391	0.083	−4.73	< 0.001	[−0.554, −0.228]	*R* ^2^ = 0.07; *F* (1, 276) = 22.36; *p* < 0.001
WI ⟶ QQ	0.266	0.268	0.052	5.21	< 0.001	[0.167, 0.370]	*R* ^2^ = 0.33; *F* (2, 275) = 68.94; *p* < 0.001
WE ⟶ QQ	−0.445	−0.314	0.036	−8.70	< 0.001	[−0.385, −0.243]	
Constant ⟶ QQ	—	3.710	0.217	17.06	< 0.001	[3.282, 4.138]	

*Note: β*, standardised coefficient; *B*, unstandardised coefficient.

Abbreviations: CI, confidence interval; QQ, quiet quitting; SE, standard error; WE, work engagement; WI, workplace incivility.

**TABLE 5:** The direct and indirect effects of workplace incivility on quiet quitting.

Effect	*B*	SE	*t*	*p*	95% CI
Direct effect of WI on QQ	0.268	0.052	5.21	< 0.001	[0.167, 0.370]
Indirect effect of WI on QQ through WE	0.123	0.030	—	—	[0.068, 0.188]
Total effect of WI on QQ	0.391	0.056	7.00	< 0.001	[0.281, 0.501]
Completely standardised indirect effect of WI on QQ	0.122	0.030	—	—	[0.067, 0.186]

*Note: N* = 278. Bootstrap confidence intervals are based on 5000 resamples; indirect effects are significant when the CI excludes zero. *B*, unstandardised coefficient; WI, workplace incivility (independent variable); WE, work engagement (mediator); QQ, quiet quitting (dependent variable).

Abbreviations: CI, confidence interval; SE, standard error.

**FIGURE 1:**
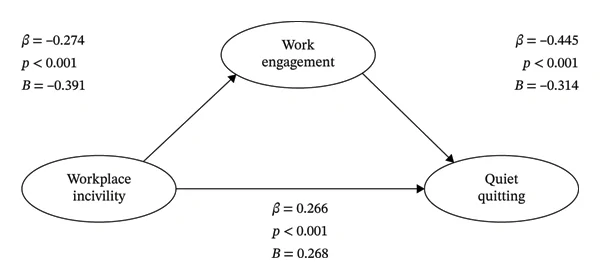
Conceptual model of the relationships among variables. *β*: standardised coefficient, *B*: unstandardised coefficient, *p* < 0.001.

Consistent with partial mediation, the direct effect of WI on QQ remained significant after WE was entered into the model (*β* = 0.266, *p* < 0.001), and the indirect effect via WE was also significant (*B* = 0.123, 95% CI [0.068, 0.188]; the interval did not include zero). As shown in Tables 4 and 5, the direct association between WI and QQ remained significant after WE was entered into the model, and the indirect association through WE was also significant, with the bootstrap confidence interval excluding zero. In practical terms, healthcare workers who reported less WI and higher WE also reported lower QQ intention.

## 4. Discussion

In this study, a detailed analysis was conducted by examining a healthcare institution to determine the impact of behaviours exhibited by colleagues and supervisors in healthcare workers’ work environments on their individual decisions regarding employment. In this context, the relationship between WI, WE and QQ was examined. The mediating effect of WE on the impact of WI on QQ was identified. The results showed a significant negative association between WI and WE; given the cross‐sectional design, this and the associations reported below should be interpreted as statistical relationships rather than proof of causal direction.

It is noted that WI is an important organisational behavioural variable that negatively affects WE [27]. It has been observed that employees experiencing higher levels of workplace courtesy report better outcomes when they feel organisationally and emotionally supported. Additionally, experiences of workplace discourtesy are associated with greater psychological distress [2]. Lee et al. [28] stated that WI and heavy workloads increase burnout, reducing job satisfaction and organisational commitment while increasing intention to leave. The importance of a culture of courtesy, zero‐tolerance policies, open communication and supportive organisational practices in preventing these problems is emphasised. Spence Laschinger et al. [29] stated that among nurses, empowerment and perceptions of supervisory rudeness were strongly associated with job satisfaction, organisational commitment and QQ, and that empowering management strategies could contribute to preventing workplace rudeness and burnout.

Galanis et al. [14] and Durmuş et al. [30] stated that WI and negative coping strategies increase the intention to QQ and resign, that work‐related stress reinforces these effects, and that positive coping strategies reduce this tendency among nurses. Accordingly, it is recommended that nursing and health administrators and policymakers take measures to reduce workplace bullying and stress factors, strengthen positive coping strategies and develop effective solutions. Moisoglou et al. [31] emphasised that levels of QQ among nurses increase in environments characterised by adverse working conditions, and that this situation negatively impacts workplace interactions.

Another finding from our study was that WE was also negatively and significantly associated with QQ. Higher WE among healthcare workers was associated with lower QQ. Recent publications indicate that WE is among the strongest negative correlates of QQ and that lower WE is linked to QQ behaviour through psychological withdrawal among employees. Higher levels of employee WE were associated with lower QQ intention, indicating that WE helps mitigate the negative effects of WI.

Hungerford et al. [32] highlighted the importance of commitment to the job, colleagues and supervisors, and noted that deficiencies in these areas can lead to disengagement from the workplace. Job commitment is a behaviour that many organisations seek. Going the extra mile is an expectation of many employers. Such disengagement behaviours, particularly in nursing, can negatively impact patients, teams, services and institutions.

In this study, the effect ranging from WI to QQ was partially mediated by WE. It appears that WE plays a role as a partial mediating variable. Accordingly, WI is indirectly and significantly associated with QQ via its association with WE. In other words, WI was associated with lower WE, which was in turn associated with greater QQ intention. In this context, the WE variable plays a mediating role by partially reducing the direct effect of WI on QQ. Smith et al. [33] noted that WI was significantly associated with higher QQ behaviour through burnout and psychological detachment among employees. They suggested that supportive nurse managers could reduce WI by shaping the work environment. In a study conducted by Nassar et al. [34] with nurses, significant relationships were identified between WI and quality of work life as well as turnover intention. However, no significant relationship was found between WI and job commitment. Based on the research findings, it is recommended that hospitals establish clear and explicit procedures for reporting rude behaviour in the workplace. In the study by Gün et al. [35], it was demonstrated that the intention to leave plays a significant role in the relationship between job burnout and QQ.

Although the cross‐sectional design of the study precludes causal inference, there are complementary mechanisms that may explain why incivility is accompanied by lower engagement and, consequently, by greater QQ intention. When interpreted within the framework of COR theory [3], the present findings gain greater explanatory power. WI depletes employees’ psychological resources; employees therefore reduce their WE in order to preserve their remaining resources, and QQ follows as a deliberate conservation strategy. Incivility can thus be conceptualised as a resource‐loss process, the decline in engagement as a direct reflection of that loss and QQ as the protective response [3, 4]. Incivility is a chronic, low‐intensity stressor whose ambiguity requires the target to expend cognitive and emotional resources on interpreting intent and managing affect; because episodes recur and are rarely resolved, depletion is cumulative. Engagement requires precisely a surplus of these resources and is therefore the first casualty, and QQ becomes a deliberate protective strategy rather than indifference. Guo et al. [36] showed that the work incivility and WE link is transmitted through perceived insider status, affective commitment and organisational identification; QQ may therefore represent a withdrawal of psychological membership before it is a withdrawal of effort.

Research shows that rude behaviour in the healthcare sector leads to poor quality of patient care. Alshehry et al. [37] stated that general rudeness in the healthcare sector and rude behaviour towards nurses negatively affect the quality of nursing care. Therefore, it is recommended that managers develop effective policies to prevent rude behaviour and strengthen reporting and timely intervention mechanisms. Othman et al. [38] reported significant relationships between QQ, perceived silent dismissal, nurse loyalty and intention to leave. Accordingly, it is recommended to strengthen nurses’ participation in management, promotion opportunities, professional practice models and mentoring programmes. According to Galanis et al. [39], the rate of QQ among nurses was 67.4%, among doctors 53.8% and among other healthcare workers 40.3%. Additionally, it was found that shift workers have higher levels of QQ compared to those working in the private sector. In light of these findings, it is emphasised that policymakers and managers must develop and implement intervention strategies at both the organisational and individual levels to prevent or reduce QQ. In a study conducted in the Greek healthcare sector, it was emphasised that management plays a critical role in reducing turnover rates to establish a sustainable and resilient healthcare workforce [40]. Furthermore, the study notes that the partial rather than full mediation observed is consistent with the common functioning of these mechanisms and that simply increasing commitment will not be sufficient; exposure to incivility itself must also be reduced.

### 4.1. Limitations

The findings of this study should be interpreted in the light of several limitations.

First, because participants were selected by convenience sampling from a single private hospital operating in Istanbul, the generalisability of the findings is limited. The results should therefore be regarded as specific to this institution and should be confirmed by multicentre studies covering different regions and different types of healthcare organisations.

Second, because all variables were measured at a single time point using self‐report methods, common method bias cannot be entirely excluded. The procedural remedies applied and the statistical checks performed indicate that common method bias did not substantially affect the findings; however, these checks do not eliminate common method bias entirely but only reduce its influence.

Third, the cross‐sectional design of the study does not permit determination of the temporal ordering of, or causal relationships among, the variables. The mediation model should therefore be interpreted not as a causal mechanism but as a theoretical model showing statistical relationships among the variables. Longitudinal and experimental studies are needed to confirm the findings.

Fourth, face‐to‐face administration in the hospital setting may increase socially desirable responding on sensitive items concerning incivility and withdrawal intentions. In future studies, the mode of administration should be recorded for each participant and measurement invariance across modes should be tested.

Fifth, the QQ Intention Scale did not perform adequately in its original seven‐item form within this sample. Two items (QQ2 and QQ7) were excluded from the analysis because they fell below the predetermined item retention criteria, and the analyses were conducted using the remaining five items. First, the excluded QQ2 item refers to restricting effort to the minimum required by the job description, which is widely regarded as the defining behavioural expression of QQ. Its removal means that the construct as measured here reflects primarily the attitudinal component of QQ rather than its behavioural component, and the findings should be interpreted accordingly. This decision improved the scale’s psychometric properties but did not alter the core findings. It is recommended that the scale be validated in samples of healthcare professionals and that items reflecting the behavioural dimension of QQ be developed in this context.

Sixth, no a priori power analysis was conducted before data collection. The sensitivity power analysis carried out after data collection indicated that the present sample had sufficient power to detect the effect sizes reported; however, conducting an a priori power analysis is recommended when planning future studies, particularly those intended to assess small effect sizes.

Taken together, these limitations indicate that the present findings are best interpreted as evidence of a theoretically coherent pattern of associations within one institution at one point in time, rather than as a demonstration of a causal chain.

## 5. Conclusion

This study contributes to the literature by examining the mediating role of WE in the relationship between WI and QQ. The findings indicate that WI was positively associated with QQ intention among healthcare workers and that WE partially mediated this association. WI was negatively associated with WE, and WE was in turn negatively associated with QQ intention. Furthermore, when we examined the quantitative findings, the total effect of WI on QQ was weaker when WE was taken into account, consistent with partial mediation. Higher levels of WE were associated with lower QQ intention, suggesting that WE may function as a protective mechanism.

The findings also indicate that focusing solely on the direct relationship between WI and QQ may be insufficient, and that the QQ intentions of healthcare workers who encounter incivility appear to be linked indirectly through WE. This is particularly noteworthy in healthcare settings, where managers’ responses to incivility and their interventions to sustain engagement may be consequential for workforce stability.

The examined model offers inferences regarding the importance of evaluating the consequences of behavioural patterns observed in organisations by healthcare institution employees and healthcare managers. It has been observed that disrespectful behaviour encountered by healthcare workers affects WE and QQ. It has been determined that managers need to take steps to create a safe working environment for employees. Identifying and reducing the factors affecting healthcare workers will facilitate increasing WE and reduce the intention to QQ. In this respect, the study provides valuable information for managers. It is important to know that controlling disrespectful behaviour in the workplace is necessary and that its consequences can have negative effects. Developing strategies to increase job commitment and reduce QQ among healthcare workers is also important in terms of improving the quality of care provided to patients. Understanding the relationships between WI, WE and QQ is a crucial step in developing management strategies in the healthcare sector. This study demonstrates the necessity of providing psychological and emotional support to healthcare workers and its positive impact on the workforce. It contains important information for managerial practices in the healthcare sector, especially for healthcare workers, and should be considered in management and strategy development processes. It is crucial for managers to develop practices, policies and strategies to mitigate the effects of negative behaviours among healthcare professionals. These measures aim to provide social and psychological support to address the problems of healthcare workers. This study is expected to contribute to the fields of human resources management and organisational behaviour.

## Author Contributions

Conceptualization, Hafize Boyacı and Selma Söyük; methodology, Hafize Boyacı; data collection, Emin Aksan; formal analysis, Hafize Boyacı; data curation, Hafize Boyacı; interpretation of findings, Hafize Boyacı and Selma Söyük; writing–original draft preparation, Hafize Boyacı, Selma Söyük and Emin Aksan; writing–review and editing, Hafize Boyacı and Selma Söyük; supervision, Hafize Boyacı and Selma Söyük.

## Funding

No funding was received for this research.

## Disclosure

Because workforce instability arising from quiet quitting may adversely affect the quality and continuity of patient care, the findings are directly relevant to healthcare service users. All authors read and approved the final version of the manuscript.

## Conflicts of Interest

The authors declare no conflicts of interest.

## Data Availability

The data that support the findings of this study are available from the corresponding author upon reasonable request. Restrictions apply to the availability of these data as they were collected under institutional ethics approval with participant confidentiality guarantees.
